# Supra-hepatic gallbladder in cirrhosis presenting as a mimic of intra-abdominal fat necrosis: Diagnostic pitfalls and clinical implications

**DOI:** 10.4102/sajr.v30i1.3484

**Published:** 2026-07-23

**Authors:** Siddhi Chawla, Binit Sureka, Mahaveer S. Rodha

**Affiliations:** 1 Department of Trauma and Emergency (Diagnostic and Interventional Radiology), All India Institute of Medical Sciences, Jodhpur, India; 2 Department of Diagnostic and Interventional Radiology, All India Institute of Medical Sciences, Jodhpur, India; 3 Department of Trauma and Emergency (Surgery), All India Institute of Medical Sciences, Jodhpur, India

**Keywords:** computed Tomography, suprahepatic gall bladder, anatomical variation, fat necrosis, ectopic gallbladder

## Abstract

Supra-hepatic gallbladder is an exceptionally rare form of ectopic gallbladder. Its identification can be particularly challenging in patients with cirrhosis, where distortion of hepatic architecture obscures expected anatomical relationships. This report describes a case of supra-hepatic gallbladder in a patient with chronic liver disease, in which surrounding inflammatory oedema mimicked intra-abdominal fat necrosis, creating a significant diagnostic dilemma on imaging.

**Contribution:**

This case underscores the importance of recognizing unusual gallbladder locations, understanding their embryological basis, and being aware of potential imaging pitfalls and complications associated with ectopic positioning.

## Introduction

Ectopic gallbladder is an uncommon developmental anomaly with several reported locations, including intrahepatic, left-sided, retrohepatic, and suprahepatic positions.^1,2^ Among these, the suprahepatic variant is exceedingly rare, with the gallbladder located along the hepatic dome between the liver and diaphragm. Recognition of this anomaly is important because it may lead to atypical imaging findings, delayed diagnosis and increased operative complexity. These difficulties are particularly relevant in cirrhosis, where altered hepatic morphology can further obscure anatomical relationships.^3^

## Ethical considerations

Ethical clearance to conduct this study was obtained from the Institutional Ethics Committee (No. AIME/JDH/T&E/2026/JUN/24).

## Case presentation

A 42-year-old woman with known chronic liver disease (CLD) presented with jaundice, upper gastrointestinal bleeding, and progressive hepatic dysfunction. Laboratory investigations revealed hyperbilirubinaemia (total bilirubin 2.95 mg/dL), hypoalbuminaemia (2.9 g/dL), mild transaminitis (aspartate aminotransferase 59.3 U/L, alanine aminotransferase 41.1 U/L), prolonged prothrombin time (17.8 s; international normalised ratio 1.46), and markedly reduced fibrinogen levels (116 mg/dL), indicating impaired hepatic synthetic function. Haematological evaluation demonstrated thrombocytopaenia (60 000/µL) and an elevated erythrocyte sedimentation rate (49 mm/hr). Reduced complement levels (C3 54.1 mg/dL, C4 6.4 mg/dL) were attributed to the underlying CLD. Renal function was preserved. Tumour markers, including alpha-fetoprotein (AFP), carcinoembryonic antigen (CEA), and cancer antigen 125 (CA-125), were within normal limits, while carbohydrate antigen 19-9 (CA19-9) was mildly elevated (45 U/mL).

Ultrasonography revealed altered hepatic echotexture with irregular nodular margins consistent with cirrhosis, along with gallbladder wall oedema measuring 4.8 mm, splenomegaly (15.8 cm), multiple perisplenic collateral vessels, and prominence of the portal vein, suggestive of portal hypertension. No ascites was identified. A small haemangioma was noted in the left hepatic lobe.

**FIGURE 1:**
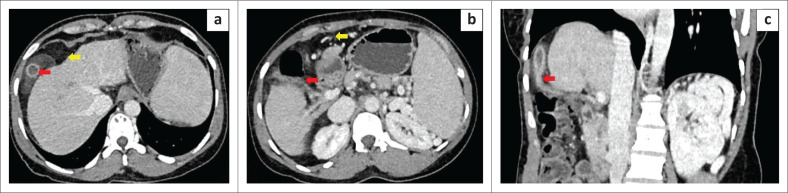
Contrast-enhanced CT images demonstrating a suprahepatic gallbladder in a patient with cirrhosis. (a) Axial image demonstrates an ectopic gallbladder (red arrow) situated along the superior surface of the right hepatic lobe with associated pericholecystic oedema. Cirrhotic liver morphology is evidenced by surface nodularity and irregular hepatic contour (yellow arrow). (b) Axial image at the level of the gallbladder fossa shows continuity of the ectopic gallbladder with the cystic duct (red arrow), while prominent omental collaterals (yellow arrow) indicate portal hypertension. (c) Oblique coronal reformatted image clearly delineates the suprahepatic position of the gallbladder (red arrow), located between the hepatic dome and the right hemidiaphragm.

To further evaluate the cause of jaundice, contrast-enhanced CT of the abdomen was performed. The CT demonstrated cirrhotic liver morphology with a nodular contour, heterogeneous enhancement, splenomegaly, and porto-systemic collaterals. Incidentally, the gallbladder was not visualised within its usual fossa. Instead, a well-defined fluid-attenuation structure was identified along the anterosuperior aspect of the right hepatic lobe, interposed between the hepatic dome and diaphragm, consistent with a suprahepatic gallbladder. Associated pericholecystic oedema and surrounding subdiaphragmatic fat stranding were present (Figure 1).

As a result of its unusual location and adjacent inflammatory changes, the lesion initially posed a diagnostic challenge, with differential considerations including a thrombosed collateral vessel, epiploic appendagitis, omental infarction, or another atypically located inflammatory process. Careful review of multiplanar reformatted images demonstrated continuity with the cystic duct, confirming its biliary origin and establishing the diagnosis of a suprahepatic gallbladder. No cholelithiasis, focal wall thickening, or gallbladder mass was identified.

The suprahepatic gallbladder was considered an incidental developmental anomaly unrelated to the patient’s presenting symptoms. The patient was managed conservatively for chronic liver disease with standard medical therapy, including rifaximin. No specific intervention was required for the ectopic gallbladder. She remained clinically stable and was discharged with advice for regular follow-up in the hepatology outpatient department for ongoing management of her CLD.

## Discussion

Suprahepatic gallbladder represents one of the rarest forms of ectopic gallbladder, with only isolated cases reported since its earliest description in the mid-twentieth century.^4,5^ It is most frequently associated with developmental abnormalities of the right hepatic lobe, particularly hypoplasia or agenesis, resulting in cranial displacement of the gallbladder fossa.^5,6,7^ Embryologically, the gallbladder arises from the caudal bud of the hepatic diverticulum during the fourth week of gestation. Its final position depends on coordinated hepatic growth and rotation, and aberrant migration or failure of descent of the gallbladder primordium is believed to underlie ectopic locations. The frequent association with right hepatic lobe anomalies supports a developmental basis rather than a simple positional variant.^5,7,8^

A variety of associated abnormalities have been described, including hepatic inversion, diaphragmatic defects, and other congenital hepatobiliary anomalies.^4,8^ In contrast, the present case occurred in a cirrhotic liver without overt right hepatic lobe agenesis or hypoplasia. Although the ectopic location was likely developmental in origin, progressive architectural distortion, capsular retraction, and segmental volume redistribution associated with cirrhosis, may have accentuated its appearance and contributed to the unusual imaging presentation. This observation broadens the spectrum of clinical settings in which a suprahepatic gallbladder may be encountered.^9^

From a radiological perspective, failure to identify the gallbladder in its orthotopic fossa should prompt a systematic search for ectopic locations. Identifying the suprahepatic variant can be particularly challenging because of its close relationship to the diaphragm, hepatic dome and subdiaphragmatic fat. In the present case, associated pericholecystic oedema and fat stranding obscured the diagnosis and initially suggested a focal inflammatory process. While previous reports have described confusion with subdiaphragmatic collections or hepatic lesions,^8^ the present case highlights an additional imaging pitfall: the combination of an ectopic location and surrounding inflammatory change can simulate intra-abdominal fat necrosis and other inflammatory conditions. The diagnostic difficulty was further compounded by the background of cirrhosis and portal hypertension, where collateral vessels and altered hepatic morphology can confound image interpretation.

Recognition of this anomaly is clinically important because ectopic gallbladders may present with atypical symptoms and delayed diagnosis of biliary pathology.^6,8^ Furthermore, abnormal peritoneal attachments may increase mobility of the gallbladder, predisposing to uncommon but potentially serious complications such as torsion, gangrene, and fistula formation.^5,8,10^ From a surgical standpoint, altered orientation of the cystic duct and cystic artery may increase the risk of intraoperative disorientation and bile duct injury, particularly during laparoscopic procedures that rely heavily on conventional anatomical landmarks.^2,3^ Rare identification of suprahepatic gallbladders on advanced imaging modalities such as fluorodeoxyglucose-positron emission tomography (FDG PET/CT) further underscores the need for radiological awareness across subspecialties.^9^

A structured imaging approach is therefore essential. When the gallbladder is not visualised in its expected location, careful evaluation of the subhepatic, intrahepatic, retrohepatic, and suprahepatic regions should be undertaken. Demonstration of a cystic structure with characteristic morphology, identification of continuity with the cystic duct on multiplanar CT reconstructions or MR cholangiopancreatography, and correlation with the biliary tree are key steps in establishing the diagnosis. Such an approach is particularly valuable in cirrhotic patients, in whom conventional anatomical landmarks may be distorted.

## Conclusion

This case illustrates a rare suprahepatic gallbladder in a patient with cirrhosis, where associated pericholecystic oedema and inflammatory changes created a significant diagnostic dilemma by mimicking other intra-abdominal inflammatory processes. Awareness of this uncommon entity, combined with meticulous evaluation of biliary anatomy on multiplanar imaging, is essential to ensure accurate diagnosis and avoid potential clinical and surgical complications.
